# Recent advances in the dissection of drought-stress regulatory networks and strategies for development of drought-tolerant transgenic rice plants

**DOI:** 10.3389/fpls.2015.00084

**Published:** 2015-02-18

**Authors:** Daisuke Todaka, Kazuo Shinozaki, Kazuko Yamaguchi-Shinozaki

**Affiliations:** ^1^Laboratory of Plant Molecular Physiology, Graduate School of Agricultural and Life Sciences, The University of Tokyo, TokyoJapan; ^2^RIKEN Center for Sustainable Resource Science, YokohamaJapan

**Keywords:** drought, stress signaling, stress tolerance, rice, transgenic engineering

## Abstract

Advances have been made in the development of drought-tolerant transgenic plants, including cereals. Rice, one of the most important cereals, is considered to be a critical target for improving drought tolerance, as present-day rice cultivation requires large quantities of water and as drought-tolerant rice plants should be able to grow in small amounts of water. Numerous transgenic rice plants showing enhanced drought tolerance have been developed to date. Such genetically engineered plants have generally been developed using genes encoding proteins that control drought regulatory networks. These proteins include transcription factors, protein kinases, receptor-like kinases, enzymes related to osmoprotectant or plant hormone synthesis, and other regulatory or functional proteins. Of the drought-tolerant transgenic rice plants described in this review, approximately one-third show decreased plant height under non-stressed conditions or in response to abscisic acid treatment. In cereal crops, plant height is a very important agronomic trait directly affecting yield, although the improvement of lodging resistance should also be taken into consideration. Understanding the regulatory mechanisms of plant growth reduction under drought stress conditions holds promise for developing transgenic plants that produce high yields under drought stress conditions. Plant growth rates are reduced more rapidly than photosynthetic activity under drought conditions, implying that plants actively reduce growth in response to drought stress. In this review, we summarize studies on molecular regulatory networks involved in response to drought stress. In a separate section, we highlight progress in the development of transgenic drought-tolerant rice plants, with special attention paid to field trial investigations.

## INTRODUCTION

Drought is inevitable. For example, the U.S. suffered an agricultural drought in 2012, in which a 12% decrease in corn production was recorded compared with the previous year (USDA, 2014). Because such decreases in crop production cause enormous economic disruption, demand for the development of drought-tolerant crops is increasing.

Rice (*Oryza sativa* L.) is one of the world’s most important cereals, with production comparable to that of wheat. In 2013, rice and wheat were cultivated in 124 and 126 countries, respectively, with corresponding worldwide production of 745 and 713 million tons (FAOSTAT). Compared with other cereal crops such as maize and wheat, rice is sensitive to decreases in soil water content because rice cultivars have been historically grown under flood irrigation conditions where the soil matric potential is zero. As a consequence, large amounts of water are required for production of rice compared with other crops. Production of 1 kg of rice seed requires 3,000 to 5,000 L of water, with less than half that amount needed for 1-kg seed production in other crops such as maize or wheat (Singh et al., 2002). Improvement of water-use efficiency during rice production should thus contribute significantly to agricultural water conservation and deserves much attention. Rice cultivars showing normal or even increased yield under drought stress conditions are expected to be closely related to those with high water-use efficiency.

Transgenic engineering approaches in plants have opened the door to the development of new cultivars with improved drought tolerance. Progress has been made in the generation of transgenic drought-tolerant rice plants. In this review, we begin with an overview of abiotic stress signaling pathways coordinated by a wide range of regulatory proteins, including key factors for the development of transgenic drought-tolerant rice plants, and then describe advances in the development of transgenic drought-tolerant rice. We also discuss growth regulatory mechanisms operating under water deficit stress conditions. Special attention is paid to transgenic rice plants showing improved drought tolerance under field conditions.

## REGULATORY MECHANISMS OF RESPONSES TO ABIOTIC STRESSES, INCLUDING DROUGHT, IN *Arabidopsis*

**FIGURE 1:**
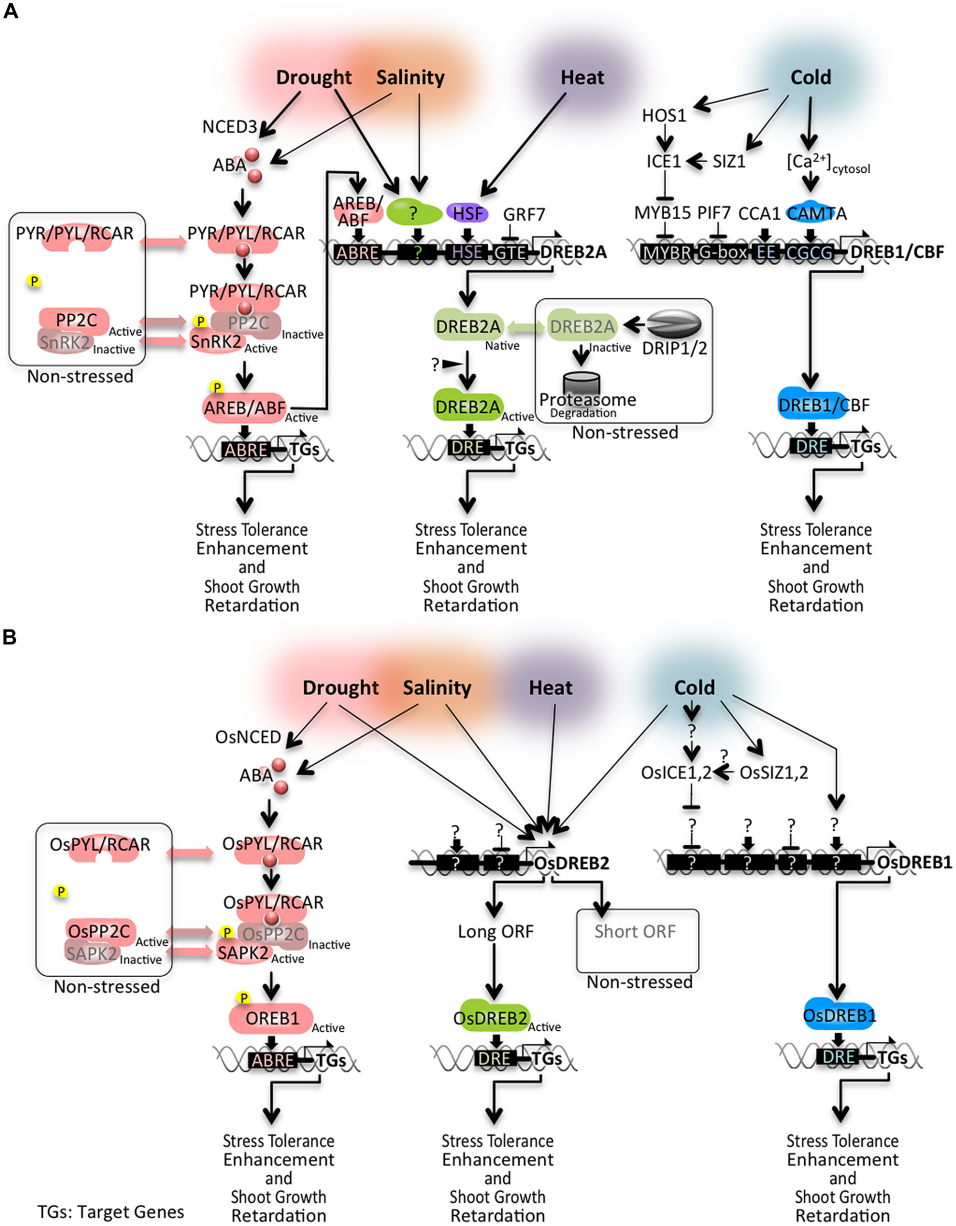
**Abiotic stress signaling networks mediated by AREB, DREB1, and DREB2-type transcription factors **(A)** in *Arabidopsis* and **(B)** in rice**.

Abiotic stresses, such as drought, high salinity, and low temperature, induce the expression of a large number of genes. The induction of these genes under stress is regulated through complex transcriptional networks (Yamaguchi-Shinozaki and Shinozaki, 2006). The key genes functioning in these transcriptional networks have been revealed by molecular studies and are important candidates for the development of transgenic plants tolerant to abiotic stress. Here, we highlight two important pathways of transcriptional networks under abiotic stress conditions in *Arabidopsis*: an abscisic acid (ABA)-dependent signaling pathway and an ABA-independent regulatory network mediated by dehydration responsive element-binding (DREB)-type transcription factors (**Figure 1A**). Numerous excellent review articles on global abiotic stress regulatory networks have previously been published (Zhu, 2002; Bartels and Sunkar, 2005; Chinnusamy et al., 2007; Hua, 2009; Thomashow, 2010; Qin et al., 2011; Deinlein et al., 2014; Golldack et al., 2014; Yoshida et al., 2014).

### THE ABA-DEPENDENT SIGNALING PATHWAY

In *Arabidopsis*, significant progress has been made in the elucidation of molecular mechanisms of transcriptional networks involved in abiotic stress response. The phytohormone ABA is a major molecule facilitating signal transduction during drought stress response. A key enzyme for ABA biosynthesis is 9-*cis*-epoxycarotenoid dioxygenase (NCED; Iuchi et al., 2001). Among five genes encoding NCED in *Arabidopsis*, expression of *NCED3* has been found to increase under water deficit conditions (Iuchi et al., 2001). A G-box recognition sequence located -2,248 bp from the *NCED3* transcriptional start site has recently been shown to be important for this gene’s expression under water deficit conditions (Behnam et al., 2013). It has also emerged that ABA intercellular transport mechanisms are important for ABA-dependent drought responses. Kuromori et al. (2014) have demonstrated that specific cells in vascular tissue synthesize ABA and transport the molecule to target cells. Bauer et al. (2013) have proposed that ABA is autonomously synthesized in guard cells.

Synthesized or transported ABA is perceived by a receptor complex, which consists of PYR (PYRABACTIN RESISTANCE)/PYL (PYR1-LIKE)/RCAR (REGULATORY COMPONENT OF ABA RESPONSE), PP2C (protein phosphatase 2C), and SnRK2 (sucrose non-fermenting 1-related protein kinase 2; Cutler et al., 2010; Raghavendra et al., 2010; Umezawa et al., 2010; Weiner et al., 2010). A suite of studies has clarified the molecular structural changes that occur during ABA perception and in the ABA signaling cascade (Cutler et al., 2010; Raghavendra et al., 2010; Umezawa et al., 2010; Weiner et al., 2010; Miyakawa et al., 2013). In the absence of ABA, PP2Cs repress the ABA signaling pathway by dephosphorylation-triggered inactivation of SnRK2s. In the presence of ABA, ABA-bound PYL/PYR/RCARs recognize and bind to PP2Cs, thereby releasing SnRK2s from PP2C-dependent negative regulation. The activated SnRKs phosphorylate downstream proteins including AREB/ABF (ABA-responsive *cis*-element binding protein/ABA-responsive *cis*-element binding factor) transcription factors. The AREB/ABF transcription factors have a bZIP domain and four conserved domains containing SnRK2 phosphorylation sites. The phosphorylated AREB/ABFs are activated and bind to the ABA-responsive *cis*-element (ABRE; PyACGTGG/TC), which is enriched in promoter regions of drought-inducible genes. AREB/ABFs function as master transcriptional activators regulating ABRE-dependent gene expression in ABA signaling under drought stress conditions.

### THE ABA-INDEPENDENT SIGNALING PATHWAY MEDIATED BY DREB2 AND DREB1/CBF

Evidence has revealed that ABA-independent signaling pathways are also important in abiotic stress response. DREB2 proteins are members of the AP2/ERF family of plant-specific transcription factors. Among the eight *DREB2* genes in *Arabidopsis*, *DREB2A* and *DREB2B* are highly induced by drought, high salinity, and heat stress, and function as transcriptional activators in the ABA-independent pathway. A negative regulatory domain has been identified in the DREB2A amino acid sequence and is reported to be involved in DREB2A protein stability (Sakuma et al., 2006a; Mizoi et al., 2012). Under non-stressed conditions, degradation of DREB2A proteins occurs via ubiquitination of DREB2A by the C3HC4 RING domain-containing proteins DRIP1 (DREB2A-interacting protein1) and DRIP2 (Qin et al., 2008; Morimoto et al., 2013). Kim et al. (2012a) have proposed that *DREB2A* expression is repressed by GRF7, a growth-regulating factor, to prevent growth inhibition under non-stressed conditions. DREB2A also plays a role in high temperature stress response and increased heat stress tolerance (Sakuma et al., 2006b). The use of HsfA1 multiple mutants has revealed that *DREB2A* expression under heat stress conditions is regulated by HsfA1 (heat shock factor A1) proteins (Yoshida et al., 2011).

DREB1/CBF transcription factors are another subfamily that regulates expression of many abiotic stress-responsive genes. DREB1/CBFs are key regulators in low-temperature stress-responsive gene expression. Transgenic *Arabidopsis* plants overexpressing *DREB1/CBF* genes have been found to improve low-temperature stress tolerance as well as drought and salinity stress tolerance (Kasuga et al., 1999; Yamaguchi-Shinozaki and Shinozaki, 2006). Several studies have reported that expression of *DREB1/CBF* genes is directly or indirectly modulated by regulatory factors such as HOS1 (Dong et al., 2006), ICE1 (Chinnusamy et al., 2003), SIZ1 (Miura et al., 2007), MYB15 (Agarwal et al., 2006), PIF7 (Kidokoro et al., 2009), CAMTA3 (Doherty et al., 2009), and a clock component (Dong et al., 2011). Recent excellent reviews by Medina et al. (2011) and Mizoi et al. (2012) are highly informative regarding DREB transcription factor proteins and the related signaling cascade in *Arabidopsis*.

## MOLECULAR RESPONSES TO DROUGHT STRESS IN RICE

In rice, more than 5,000 genes are up-regulated and more than 6,000 are down-regulated by drought stress (Maruyama et al., 2014). A comparison between rice and *Arabidopsis* by Maruyama et al. (2014) demonstrated that different metabolites are accumulated under abiotic stress conditions. For example, high expression levels of genes encoding isocitrate lyase and malate synthase in the glyoxylate cycle along with glucose accumulation under abiotic stress conditions were observed in rice, but not in *Arabidopsis*. Additionally, reduced expression of the cytochrome P450 735A gene was correlated with decreased cytokinin levels in rice, but not in *Arabidopsis*. Similar comprehensive analyses of drought stress-responsive genes, proteins, and metabolites in rice have been performed as follows. Wang et al. (2011a) identified 5,284 drought stress-responsive genes. Lenka et al. (2011) compared drought-responsive genes in *indica* rice genotypes having contrasting drought tolerances. Up-regulation of the α-linolenic acid metabolic pathway was observed in the drought-tolerant genotype. Degenkolbe et al. (2009) also investigated comprehensive expression profiles of drought-responsive genes in both drought-sensitive and drought-tolerant rice genotypes. They found that senescence-related degradation processes and expression of photosynthesis-related genes were reduced in drought-tolerant cultivars compared with those in drought-sensitive ones. Using the comprehensive expression data, the authors also identified marker transcripts for selection of drought tolerance in a range of rice germplasm resources through integrated analyses of gene expression and stress tolerance (Degenkolbe et al., 2013). The marker transcripts showed a significant correlation between expression level and tolerance under drought stress conditions. One of the markers was a gene for cytosolic fructose-1,6-bisphosphatase, an enzyme that catalyzes a highly regulated step in C-metabolism. Ray et al. (2011) have reported that genes responsive to drought stress conditions significantly overlap with those expressed during panicle and seed development. Shu et al. (2011) performed integrated analyses of two-dimensional electrophoresis – mass spectrometry – mass spectrometry, cDNA microarray, and gas chromatography – mass spectrometry data from rice seedlings after drought stress treatment to, respectively, determine protein expression levels, gene expression levels, and metabolite contents. The authors speculated that energy conversion from carbohydrates and/or fatty acids to amino acids increased under drought stress conditions. Epigenetic research on drought response in rice has been also reported. Wang et al. (2011b) examined drought-induced genome-wide DNA methylation and its association with drought tolerance. Zong et al. (2013) investigated the genome-wide distribution pattern of histone H3 lysine 4 tri-methylation and found that methylation levels were positively correlated with expression levels of some of the evaluated drought-responsive genes.

While these large data sets have provided much information on drought responses in rice, studies on associated signaling cascades have been limited. Evidence indicates that *O. sativa NCED* transcripts are up-regulated along with ABA accumulation under drought stress conditions (Maruyama et al., 2014), and that a core ABRE sequence in the promoter regions of drought-inducible genes is enriched in rice, *Arabidopsis* and soybean (Maruyama et al., 2012). These results suggest that the ABA-dependent signaling pathway in rice is activated by drought stress, similar to that of *Arabidopsis* and other plant species. He et al. (2014) determined the crystal structure of the ABA–OsPYL2–OsPP2C06 complex and suggested that the complex has the potential to be an ABA receptor in rice. Kim et al. (2012b) identified a rice ABA signaling unit composed of OsPYL/RCAR5, OsPP2C30, SAPK2, and OREB1 for ABA-dependent gene regulation. They have reported that OsPYL/RCAR5 functions as a positive regulator of abiotic stress-responsive gene expression and that transgenic rice plants overexpressing *OsPYL/RCAR5* have improved drought tolerance. The OREB1 bZIP-type transcription factor, which is ortholog to *Arabidopsis* AREB, has been shown to regulate the ABA-dependent pathway in rice (Chae et al., 2007; Hong et al., 2011).

Experimental evidence has demonstrated that rice DREB transcription factors also function as important regulators in ABA-independent drought responses. The rice genome contains five DREB2-type genes, two of which—*OsDREB2A* and *OsDREB2B*—are up-regulated by abiotic stress (Matsukura et al., 2010). Transgenic rice plants overexpressing these two genes have been found to have increased drought tolerance (Chen et al., 2008; Cui et al., 2011). *OsDREB2B* generates two forms of the transcripts, *OsDREB2B1* and *OsDREB2B2*. *OsDREB2B1* encodes a non-functional protein (Short ORF in **Figure 1B**). *OsDREB2B2* contains a coding region with AP2/ERF DNA binding domain (Long ORF in **Figure 1B**). *OsDREB2B2* transcripts were accumulated by heat, cold, drought, and high salinity stress treatments, while *OsDREB2B1* transcripts were not changed except for cold stress. These results suggest that OsDREB2B2 plays an important role in the abiotic stress response of rice through the alternative splicing regulatory system (Matsukura et al., 2010). Expression of *OsDREB1A* and *OsDREB1B* is up-regulated by low-temperature stress (Dubouzet et al., 2003), while expression of *OsDREB1F* and *OsDREB1G* is increased by water deficit stress (Chen et al., 2008; Wang et al., 2008). In contrast to other DREB1-type genes in rice, OsDREB1F likely participates as a regulatory factor in the ABA-dependent pathway. Transgenic rice plants overexpressing these genes also exhibit increased drought tolerance. It has been reported that OsICE1, OsICE2, OsSIZ1, and OsSIZ2 are involved in the cold stress signaling pathway that regulates *OsDREB1B* expression (Park et al., 2010; Nakamura et al., 2011).

## TRANSGENIC RICE PLANTS THAT ENHANCE DROUGHT STRESS TOLERANCE

Genetic engineering has opened the door to the development of new cultivars with improved drought stress tolerance. Reports on transgenic rice plants that show increased drought stress tolerance are accumulating. A selected list of transgenic rice plants, which includes information on transgenes and promoters used for the transformations as well as plant stress tolerance and growth performance, is given in **Table 1**.

**Table 1:** Selective transgenic rice plants with improved drought stress tolerance.

	Construct used for transformation			Type of transgenic engineering	Performance			
Gene function	Gene name	Gene source	Promoter		Stress tolerance	Field trial	Growth trait	Reference
**Transcription factor**
bHLH	*OsbHLHU8*	Rice	Cytochrome *c*	COE*^1^	D↑*^5^	–	Not mentioned	Seo et al. (2011)
bZIP	*OsbZIP23*	Rice	Ubiquitin	COE	D↑, S↑*^6^	–	Hypersensitive to shoot growth inhibition by exogenous ABA	Xiang et al. (2008)
bZIP	*OsbZIP46*	Rice	Ubiquitin	COE	D↑, O↑*^7^	–	Hypersensitive to shoot growth inhibition by exogenous ABA	Tang et al. (2012)
bZIP	*OsbZIP71*	Rice	35S	COE	D↑, S↑,O↑	–	Not mentioned	Liu et al. (2014)
bZIP	*OsbZIP71*	Rice	RD29A	SIE*^2^	O↑	–	Not mentioned	Liu et al. (2014)
bZIP	*OsbZIP16*	Rice	Actin	COE	D↑	–	Hypersensitive to shoot growth inhibition by exogenous ABA	Chen et al. (2012)
bZIP	*ABF3*	*Arabidopsis*	Ubiquitin	COE	D↑	–	Normal shoot growth and seed production under control conditions	Oh et al. (2005)
AP2/ERF	*OSDERF1*	Rice	Actin	CRNAi*^3^	D↑	–	Not mentioned	Wan et al. (2011)
AP2/ERF	*OsERF4a*	Rice	Cytochrome *c*	COE	D↑	–	Increase in shoot growth under control conditions	Joo et al. (2013)
AP2/ERF	*OsERF4a*	Rice	Ai	AIE*^4^	D↑	–	Increase in shoot growth under control conditions	Joo et al. (2013)
AP2/ERF	*OsERF10a*	Rice	Cytochrome *c*	COE	D↑	–	Not mentioned	Joo et al. (2013)
AP2/ERF	*TSRF1*	Tomato	35S	COE	D↑, O↑	–	Increases in seedling weight and root length under drought conditions	Quan et al. (2010)
AP2/ERF	*JERF3*	Tomato	35S	COE	D↑, O↑	–	Normal shoot growth under control conditions	Zhang et al. (2010)
AP2/ERF	*OsDREB2A*	Rice	4ABRC	SIE	D↑, S↑	–	Increases in plant height and effective tillers under drought conditions	Cui et al. (2011)
AP2/ERF	*OsDREB2B*	Rice	4ABRC	SIE	D↑, S↑	–	Increases in plant height and effective tillers	Cui et al. (2011)
AP2/ERF	*HARDY*	*Arabidopsis*	35S	COE	D↑	–	Increases in shoot growth under control conditions and in root growth under drought conditions	Karaba et al. (2007)
AP2/ERF	*ZmCBF3*	Maize	Ubiquitin	COE	D↑, S↑, C↑*^8^	–	Normal yield under control conditions, hypersensitive to root growth inhibition by exogenous ABA	Xu et al. (2011)
AP2/ERF	*CBF3/DREB1A*	*Arabidopsis*	Ubiquitin	COE	D↑, S↑	–	Normal shoot growth and seed production under non-stressed conditions	Oh et al. (2005)
AP2/ERF	*CBF3/DREB1A*	*Arabidopsis*	RD29A	SIE	D↑	–	Increase in yield under drought conditions	Datta et al. (2012)
AP2/ERF	*CBF3/DREB1A*	*Arabidopsis*	HVA22	SIE	D↑	Done	Increase in yield under drought conditions	Xiao et al. (2009)
AP2/ERF	*OsDREBIA*	Rice	Ubiquitin	COE	D↑, S↑, C↑	–	Shoot growth retardation under control conditions	Ito et al. (2006)
AP2/ERF	*OsDREBIB*	Rice	Ubiquitin	COE	D↑, S↑, C↑	–	Shoot growth retardation under control conditions	Ito et al. (2006)
AP2/ERF	*CBF3/DREB1A*	*Arabidopsis*	Ubiquitin	COE	D↑, S↑, C↑	–	Shoot growth retardation under control conditions	Ito et al. (2006)
AP2/ERF	*CBF1/DREB1B*	*Arabidopsis*	Ubiquitin	COE	D↑, S↑, C↑	–	Shoot growth retardation under control conditions	Ito et al. (2006)
AP2/ERF	*CBF2/DREB1C*	*Arabidopsis*	Ubiquitin	COE	D↑, S↑, C↑	–	Shoot growth retardation under control conditions	Ito et al. (2006)
AP2/ERF	*HvCBF4*	Barley	Ubiquitin	COE	D↑, S↑, C↑	–	Normal shoot growth under control conditions	Oh et al. (2007)
AP2/ERF	*CBF2/DREB1C*	*Arabidopsis*	Lip9	SIE	D↑	–	Increase in dry weight under drought conditions	Ishizaki et al. (2012)
AP2/ERF	*AP37*	Rice	OsCcI	COE	D↑, S↑, C↑	Done	Normal shoot growth under control conditions	Oh et al. (2009)
Homeodomain	*EDT1/HDG11*	*Arabidopsis*	Actin	COE	D↑	Done	Increases in shoot, root, and seed production under drought conditions	Yu et al. (2013)
Homeodomain	*Zmhdz10*	Maize	Ubiquitin	COE	D↑, S↑	–	Hypersensitive to shoot growth inhibition by exogenous ABA	Zhao et al. (2014)
MYB	*OsMYB2*	Rice	Ubiquitin	COE	D↑, S↑, C↑	–	Hypersensitive to shoot growth inhibition by exogenous ABA	Yang et al. (2012)
NAC	*OsNAC5*	Rice	RCc3	RSE*^10^	D↑	Done	Increases in yield and root diameter under drought conditions	Jeong et al. (2013)
NAC	*OsNAC9/SNAC1*	Rice	RCc3	RSE	D↑	Done	Increases in yield and root diameter under drought conditions	Redillas et al. (2012)
NAC	*OsNAC10*	Rice	RCc3	RSE	D↑	Done	Increases in yield and root diameter under drought conditions	Jeong et al. (2010)
NAC	*OsNAC6*	Rice	Ubiquitin	COE	D↑	–	Shoot growth retardation under control conditions	Nakashima et al. (2007)
NAC	*SNAC1*	Rice	35S	COE	D↑, S↑	Done	Hypersensitive to shoot growth inhibition by exogenous ABA, increase in yield under drought conditions, normal shoot growth under control conditions	Hu et al. (2006)
WRKY	*OsWRKY30*	Rice	35S	COE	D↑	–	Not mentioned	Shen et al. (2012)
Zinc finger	*ZFP182*	Rice	35S	COE	D↑, S↑, C↑	–	Similar morphological traits under control conditions	Huang et al. (2012)
Zinc finger	*ZFP245*	Rice	35S	COE	D↑, C↑	–	Hypersensitive to shoot growth inhibition by exogenous ABA, normal shoot growth under control conditions	Huang et al. (2009)
Zinc finger	*ZFP252*	Rice	35S	COE	D↑, S↑	–	Not mentioned	Xu et al. (2008)
Zinc finger	*ZAT10*	*Arabidopsis*	Actin	COE	D↑	Done	Increases in yield under drought conditions	Xiao et al. (2009)
Zinc finger	*ZAT10*	*Arabidopsis*	HVA22	SIE	D↑	Done	Increases in yield under drought conditions	Xiao et al. (2009)
**Kinases**
Calcium-dependent protein kinase	*OsCPK4*	Rice	Ubiquitin	COE	D↑, S↑	–	Not mentioned	Campo et al. (2014)
Calcium-dependent protein kinase	*OsCDPK1*	Rice	Ubiquitin	COE	D↑	–	Decreases in plant height and grain size under control conditions	Ho et al. (2013)
Calcium-dependent protein kinase	*OsCDPK7*	Rice	35S	COE	D↑, S↑, C↑	–	Normal growth under control conditions	Saijo et al. (2000)
Calcineurin B-like protein interacting protein kinase	*OsCIPK12*	Rice	Ubiquitin	COE	D↑	–	Normal growth under control conditions	Xiang et al. (2007)
Recetor-like kinase	*OsSIKI*	Rice	35S	COE	D↑, S↑	–	Normal growth under control conditions	Ouyang et al. (2010)
Recetor-like kinase	*OsSIK2*	Rice	35S	COE	D↑, S↑	–	Decrease in shoot growth under control conditions	Chen et al. (2013)
MAP kinase kinase kinase	*NPK1*	*Arabidopsis*	Actin	COE	D↑	Done	Increases in yield under drought conditions	Xiao et al. (2009)
MAP kinase kinase kinase	*NPK1*	*Arabidopsis*	HVA22	SIE	D↑	Done	Increases in yield under drought conditions	Xiao et al. (2009)
**Phytohormones**
ABA receptor	*OsPYL/RCA R5*	Rice	Ubiquitin	COE	D↑, S↑	–	Hypersensitive to shoot growth inhibition by exogenous ABA, decrease in yield under control conditions	Kim et al. (2014)
Molybdenum cofactor sulfurase	*LOS5/ABA3*	*Arabidopsis*	Actin	COE	D↑	Done	Increases in yield under drought conditions	Xiao et al. (2009)
Molybdenum cofactor sulfurase	*LOS5/ABA3*	*Arabidopsis*	HVA22	SIE	D↑	Done	Increases in yield under drought conditions	Xiao et al. (2009)
Auxin eﬄux carrier	*OsPIN3t*	Rice	35S	COE	O↑	–	Increase in root growth under control conditions	Zhang et al. (2012)
Isopentenyl-transferase	*IPT*	*Agrobacterium*	SAPK	SMIE*^11^	D↑	–	Normal growth under control conditions	Peleg et al. (2011)
β-catotene Hydroxylase	*DSM2*	Rice	35S	COE	D↑	–	Increases in spikelet fertility under drought conditions	Du et al. (2010)
**LEA proteins**
Group 3 LEA protein	*HVA1*	Barley	Actin	COE	D↑, S↑	–	Normal growth under control conditions	Xu et al. (1996)
Group 3 LEA protein	*OsLEA3-2*	Rice	2X35S	COE	D↑, S↑	–	Normal growth under control conditions	Duan and Cai (2012)
Group 3 LEA protein	*OsLEA3-1*	Rice	35S	COE	D↑	Done	Normal grain yield under control conditions	Xiao et al. (2007)
Group 3 LEA protein	*OsLEA3-1*	Rice	HVA1-like	SIE	D↑	Done	Normal grain yield under control conditions	Xiao et al. (2007)
**Others**
Lipid transfer protein	*OsDIL*	Rice	Ubiquitin	COE	D↑	–	Increases in shoot growth under osmotic stress	Guo et al. (2013)
Heat shock protein	*OsHsp17.0*	Rice	35S	COE	D↑, S↑	–	Increases in shoot and root growth under osmotic stress	Zou et al. (2012)
Heat shock protein	*OsHsp23.7*	Rice	35S	COE	D↑, S↑	–	Increases in shoot and root growth under osmotic stress	Zou et al. (2012)
*myo*-lnositol oxygenase	*OsMlOX*	Rice	35S	COE	O↑	–	Increases in shoot growth under osmotic stress	Duan et al. (2012)
Ornithine δ-amino-transferase	*OsOAT*	Rice	Ubiquitin	COE	D↑, O↑	Done	Increases in shoot growth under osmotic stress	You et al. (2012)
His phosphotransfer protein	*OsAHP*	Rice	Ubiquitin	CRNAi	O↑	–	Decrease in internode length and increase in root growth under control conditions	Sun et al. (2014)
RING domain-containing protein	*OsRDCPl*	Rice	35S	COE	D↑	–	Not mentioned	Bae et al. (2011)
Ribosome-inactivating protein	*OsRIP18*	Rice	35S	COE	D↑, S↑	–	Not mentioned	Jiang et al. (2012)
RING-finger containing E3 ligase	*OsSDIRI*	Rice	Ubiquitin	COE	D↑	–	Normal growth at an adult stage under control conditions	Gao et al. (2011)
DNA and RNA helicase and ATPase	*OsSUV3*	Rice	35S	COE	O↑, S↑	–	Increases in shoot and root growth under high salinity	Tuteja et al. (2013)
Trehalose-6-phosphate synthase	*OsTPSI*	Rice	Actin	COE	D↑,S↑,C↑	–	Not mentioned	Li et al. (2011)
RNA binding protein	*GRP2*	*Arabidopsis*	Ubiquitin	COE	D↑	–	Normal shoot growth under control conditions	Yang et al. (2014)
RNA binding protein	*GRP7*	*Arabidopsis*	Ubiquitin	COE	D↑	–	Normal shoot growth under control conditions	Yang et al. (2014)
Harpin	*hrf1*	*Xanthomonas*	35S	COE	D↑	–	Not mentioned	Zhang et al. (2011)
Farnesyltransferase/squalene synthase	*SQS*	Maize	Ubiquitin	CRNAi	D↑	–	Normal growth under control conditions and no inhibition of seedling growth by ABA treatment	Manavalan et al. (2012)
14-3-3 protein	*ZmGF14-6*	Maize	Ubiquitin	COE	D↑	–	Decreases in shoot growth and seed production under control conditions	Campo et al. (2012)
14-3-3 protein	*GF14c*	Rice	Ubiquitin	COE	D↑	–	Not mentioned	Ho et al. (2013)
Arginine decarboxylase	*Dadc*	*Datura*	Ubiquitin	COE	D↑	–	Normal growth under control conditions	Capell et al. (2004)
Manganese superoxide dimutase	*MnSOD*	Pea	SWPA2	SIE	O↑	–	Not mentioned	Wang et al. (2005)
Ski-interacting protein	*OsSKlPa*	Rice	Actin	COE	O↑	–	Normal growth under control conditions, less growth inhibition under ABA, salinity and osmotic stress condition;	Hou et al. (2009)

*^1^COE, constitutively overexpression; *^2^SIE, stress-inducible expression; *^3^CRNAi, constitutively triggering RNA interference; *^4^AIE, ABA-inducible expression; *^5^D↑, enhanced drought stress tolerance; *^6^S↑, enhanced salinity stress tolerance; *^7^O↑, enhanced osmotice stress tolerance; *^8^C↑, enhanced cold stress tolerance; *^10^RSE, root specific expression; *^11^SMIE, stress-and maturation-induced promoter.

### bZIP-TYPE TRANSCRIPTION FACTORS

OsbZIP23, which is closely related to the *Arabidopsis* homologs ABF/AREB, is a major regulator of ABA-dependent pathways (Xiang et al., 2008). In the study of Xiang et al. (2008), *OsbZIP23*-overexpressing rice plants showed increased sensitivity to ABA at both germination and post-germination stages. The transgenic plants also exhibited enhanced tolerance to drought and salinity stresses. A transactivation assay indicated that OsbZIP23 functions as a transcriptional activator, with two regions of the OsbZIP23 amino acid sequence—at N- (1–59) and C- (210–240) termini—important for transcriptional activation. Microarray analysis detected hundreds of downstream genes of OsbZIP23 with diverse functions. These downstream genes included genes encoding stress-related transcription factors, protein kinases, dehydrins, and LEA proteins.

OsbZIP46 is a member of the subfamily that includes OsbZIP23. A transactivation assay conducted by Tang et al. (2012) revealed that an internal amino acid sequence (residues 122–219) of the OsbZIP46 protein had a negative affected on transactivation activity. A constitutively active form of OsbZIP46 (OsbZIP46CA1) was developed by deletion of this internal region. Transgenic rice plants overexpressing OsbZIP46CA1 showed increased drought tolerance. Microarray analysis was then used to detect up- or down-regulated genes in the *OsbZIP46CA1*-overexpressing transgenic rice plants. These differentially regulated genes were largely different from the OsbZIP23 downstream genes, suggesting that OsbZIP46CA1 regulates a different set of genes than does OsbZIP23. Although plant height under non-stressed conditions did not appear to differ between overexpressors and control plants, exogenous ABA application drastically decreased plant height of *OsbZIP46CA1* overexpressors. Similar growth inhibition was observed in *OsbZIP23* overexpressors. These results suggest that the ABA-dependent signaling pathway mediated by OsbZIP23 or OsbZIP46 is closely related to growth retardation mechanisms under drought stress conditions. This relationship implies that growth of transgenic rice plants overexpressing *OsbZIP23* or *OsbZIP46CA1* is decreased under drought stress conditions, even though transgenic plants exhibit increased stress tolerance.

*Arabidopsis* ABF3 belongs to the same bZIP subfamily as OsbZIP46 and OsbZIP23. Transgenic *Arabidopsis* plants overexpressing *ABF3* show improved drought tolerance (Kang et al., 2002). Under non-stressed conditions, overexpressors are morphologically identical to control plants. Transgenic rice plants overexpressing *ABF3* have also been found to exhibit improved drought tolerance without growth inhibition (Oh et al., 2005).

OsbZIP16 and OsbZIP71 are classified into group IV of the rice bZIP subfamily, which is different than the *Arabidopsis* ABF/AREB subfamily (Nijhawan et al., 2008). Transgenic rice plants overexpressing OsbZIP16 show improved drought tolerance and increased growth inhibition under exogenous ABA treatment (Chen et al., 2012), while OsbZIP71-overexpressing plants under the 35S promoter or a stress-inducible RD29A promoter exhibit improved drought, salt, and osmotic stress tolerance (Liu et al., 2014). Overexpression of these genes seems not to change plant architecture under non-stressed conditions.

These reports suggest that the bZIP-type transcription factors involved in the ABA signaling pathway are potentially useful for transgenic engineering to develop rice cultivars with enhanced drought tolerance. This notion is supported by the fact that ABA content in rice is increased by drought stress (Maruyama et al., 2014) and the finding that the ABRE *cis*-element is enriched in the promoter regions of drought-responsive genes in rice (Maruyama et al., 2012).

### AP2/ERF-TYPE TRANSCRIPTION FACTORS

As mentioned above, the *Arabidopsis DREB1A* gene is a key regulator of abiotic stress response. At least three independent research groups have developed *DREB1A*-overexpressing transgenic rice plants. Oh et al. (2005) reported that overexpression of *DREB1A* driven by the ubiquitin promoter in rice plants enhances tolerance to drought and salinity stresses without growth retardation under non-stressed conditions. They observed up-regulated expression of several genes, including those responsive to stress, in transgenic rice plants. Datta et al. (2012) developed transgenic rice plants expressing *DREB1A* under the control of the stress-inducible *RD29* promoter. The yield of the transgenic rice plants under drought stress conditions was increased compared with that of non-transgenic plants. Ito et al. (2006) also reported that transgenic rice plants overexpressing *DREB1A* with the ubiquitin promoter showed enhanced tolerance to drought, cold, and salinity stresses. Elevated contents of osmoprotectants such as free proline and soluble sugars were also observed. A microarray analysis detected up-regulated genes in the transgenic rice plants; among the uncovered genes were genes for α-amylase and dehydrins, which were different from those identified by Oh et al. (2005). Unlike Oh et al. (2005), Ito et al. (2006) found that shoot growth retardation occurred in the transgenic plants under non-stressed conditions. Ito et al. (2006) also developed transgenic rice plants overexpressing *DREB1A* homologs, *Arabidopsis* paralogs *DREB1B* and *DREB1C* and rice orthologs *OsDREB1A* and *OsDREB1B* under the control of the ubiquitin promoter. These overexpressors showed enhanced tolerance to drought, salinity, and low temperature and displayed reduced growth under non-stressed conditions. Ishizaki et al. (2012) introduced *Arabidopsis DREB1C* into the upland rice cultivar NERICA1, an interspecific hybrid between stress-resistant *O. glaberrima* Steud. and high-yield *O. sativa*. The transgenic rice plants showed improved survival under drought stress conditions. *HvCBF4*, a member of the *Arabidopsis* DREB1A subfamily, has been isolated from barley as a low-temperature responsive gene. Overexpression of *HvCBF4* was found to enhance tolerance to drought, salinity, and low temperature while shoot growth was unaffected (Oh et al., 2007). ZmCBF3, a maize AP2/ERF-type transcription factor, is also a member of the *Arabidopsis* DREB1A subfamily. In a study by Xu et al. (2011), overexpression of *ZmCBF3* in transgenic rice plants enhanced tolerance to drought, salinity, and low-temperature stresses. Yields under control conditions were unchanged in the overexpressors compared with those in non-transgenic plants.

As mentioned above, the physiological role and molecular function of *Arabidopsis* DREB2A in abiotic stress responses have been vigorously studied (Mizoi et al., 2012). *Arabidopsis* DREB2A is widely recognized as a master regulator of both drought and heat stress responses and has a high potential to enhance drought and heat stress tolerance (Mizoi et al., 2012). The DREB2 regulatory mechanism seems to be well conserved in various plant species. In rice, there are five DREB2 family genes: *OsDREB2A*, *OsDREB2B*, *OsDREB2C*, *OsDREB2E*, and *OsABI4*. Transgenic *Arabidopsis* plants overexpressing *OsDREB2B* are reported to show increased expression of DREB2A target genes and enhanced tolerance to drought and heat stresses (Matsukura et al., 2010). Transgenic rice plants overexpressing *OsDREB2B* have also been found to increase drought tolerance (Chen et al., 2008). Overexpression of *OsDREB2A* under the control of an ABA-responsive promoter in rice plants increased contents of soluble sugars and proline at the seedling stage, resulting in increases in osmotic and salinity stress tolerance (Cui et al., 2011). The transgenic rice plants exhibited increased plant height and effective tiller numbers at the reproductive stage following drought treatment.

In addition to DREB1A and DREB2 subfamily members, several AP2/ERF-type and AP2/ERF-like transcription factors have been used to develop transgenic rice plants with enhanced abiotic stress tolerance. The *Arabidopsis HARDY* gene is an AP2/ERF-like transcription factor that enhances drought and salinity stress tolerance (Karaba et al., 2007). Transgenic rice plants overexpressing *HARDY* show enhanced photosynthetic assimilation and reduced transpiration, leading to increased shoot and root biomass. OsDERF1 is a protein that directly interacts with the GCC box in the promoter regions of *OsERF3* and *OsAP2-39* (Wan et al., 2011). Knockdown of *OsDERF1* increases ethylene biosynthesis and drought tolerance, suggesting that OsDERF1 modulates drought response via ethylene production. Transgenic rice plants overexpressing *OsERF3* show decreased drought tolerance (Zhang et al., 2013). In contrast, Joo et al. (2013) have reported that overexpression of OsERF3/OsERF4a decreases expression levels of a repressor involved in defense responses, leading to increased drought tolerance and seedling shoot growth. Overexpression of a tomato ERF gene, *TSRF1*, was found to improve osmotic and drought tolerance without growth retardation in rice seedlings. The improvement was attributed to corresponding increases in the expression of genes encoding MYC and MYB-type transcription factors and genes related to ABA synthesis, proline synthesis, and photosynthesis (Quan et al., 2010). Another tomato ERF gene, *JERF3*, has also been observed to increase drought tolerance when overexpressed in transgenic rice plants (Zhang et al., 2010). The studied transgenic rice plants showed higher contents of soluble sugars and proline compared with those of non-transgenic plants. Transgenic rice plants overexpressing *AP37*, a rice AP2/ERF transcription factor, showed enhanced tolerance to drought, cold and high salinity stresses (Oh et al., 2009). Microarray analysis identified *AP37* downstream genes, which included genes for PHD zinc finger and iron transporter.

Among the AP2/ERF transcription factor genes described here, *DREB1/CBF* genes have been widely used for the development of drought-tolerant transgenic crops. These developed crops include chrysanthemum (Hong et al., 2006), peanut (Bhatnagar-Mathur et al., 2013), soybean (Polizel et al., 2011), tobacco (Kasuga et al., 2004), tomato (Hsieh et al., 2002), tall fescue (Zhao et al., 2007), and wheat (Pellegrineschi et al., 2004).

### NAC-TYPE TRANSCRIPTION FACTORS

NAC family proteins function in a wide variety of developmental processes and abiotic stress responses (Nakashima et al., 2012; Nuruzzaman et al., 2013). Constitutive overexpression of *OsNAC6* in rice plants was observed to increase tolerance to drought and salinity stresses (Nakashima et al., 2007). The transgenic rice plants showed decreased shoot growth under non-stressed conditions. When a stress-inducible promoter was used for the transgene expression, the transgenic plants showed normal growth under non-stressed conditions and improved salinity stress tolerance. In the same study, microarray analysis revealed OsNAC6 downstream genes including stress-related genes. The transgenic rice plants constitutively overexpressing *OsNAC6* also showed enhanced tolerance to blast disease, suggesting that OsNAC6 can act as a transcriptional regulator in both biotic and abiotic stress responses in rice.

In a study by Hu et al. (2006), *SNAC1* was demonstrated to be predominantly expressed in guard cells under drought conditions. Transgenic rice plants overexpressing *SNAC1* showed reduced water loss due to increased stomatal closure and enhanced expression of a large number of stress-related genes. As mentioned in the following section, the transgenic rice plants exhibited enhanced drought tolerance during field trials.

Transgenic rice plants overexpressing *OsNAC10* under the control of a root-specific promoter showed thicker roots and higher grain yields than those of control plants under drought stress conditions (Jeong et al., 2010). An accompanying microarray analysis identified various downstream genes, including *P450*, *Zn-finger*, *HAK5*, *2OG-Fe(II)*, *NCED*, *NAC*, and *KUP3* (Jeong et al., 2010). Similar to *OsNAC10* overexpressors, transgenic rice plants overexpressing *OsNAC5* (Jeong et al., 2013) and *OsNAC9/SNAC1* (Redillas et al., 2012) under root-specific promoter control have been shown to have thicker roots and higher grain yields than control plants under drought stress conditions. The microarray analysis of Jeong et al. (2013) identified 62 downstream genes of *OsNAC5*, including *NCED*, *calcium-transporting ATPase*, *germin-like protein*, and *meristem protein 5*. Only 17 of these downstream genes were up-regulated in *OsNAC10*-overexpressing transgenic rice plants (Jeong et al., 2013). With respect to *OsNAC9/SNAC1* overexpressors, identified downstream genes included *NCED* and *calcium-transporting ATPase* (Redillas et al., 2012). In addition to stress-responsive genes, OsNAC family downstream genes included genes involved in cell growth and development, suggesting that the OsNAC family is involved in regulatory mechanisms of stress responses and developmental processes. Song et al. (2011) observed that accumulations of proline and soluble sugars in *OsNAC5* overexpressors were higher than those of non-transgenic plants. Takasaki et al. (2010) have reported that OsNAC5 functions as a transcriptional activator and up-regulates expression of some stress-responsive genes in *OsNAC5* overexpressors. These authors also detected dimerization of OsNAC5 with OsNAC5, OsNAC5 with OsNAC6, and OsNAC5 with SNAC1.

### OTHER TRANSCRIPTION FACTORS

Other transcription factors have also been applied in the successful development of transgenic rice plants with enhanced drought tolerance. Some of these plants have shown increased ABA sensitivity. *Arabidopsis EDT1/HDG11* encodes a homeodomain-leucine zipper transcription factor, which is likely involved in reproductive development (Yu et al., 2008). Overexpression of *EDT1/HDG11* in rice plants has been found to enhance root development, reduce stomatal density, and increase water-use efficiency (Yu et al., 2013). In the cited study, levels of ABA, proline and soluble sugars and activities of reactive oxygen species (ROS)-scavenging enzymes under drought stress conditions were higher in transgenic rice plants than in non-transgenic ones. Global gene expression analysis showed that stress-responsive genes, including *SNAC1*, *SNAC2*, *OsbZIP23,* and *OsNCED3,* were up-regulated in the transgenic rice plants. Under drought stress conditions, the transgenic rice plants exhibited higher grain yields compared with non-transgenic plants. Zhao et al. (2014) isolated a maize homeodomain-leucine zipper transcription factor gene, *Zmhdz10*, and generated transgenic rice plants overexpressing this gene. Overexpression of *Zmhdz10* enhanced tolerance to drought and salinity stresses and increased growth inhibition under exogenous ABA treatments. In another study, a rice R2R3-type MYB gene, *OsMYB2*, was overexpressed in rice plants (Yang et al., 2012). The transgenic overexpressors showed enhanced tolerance to drought, salinity and low-temperature stresses, and normal growth rates under non-stressed conditions. Exogenous ABA treatment resulted in greater growth inhibition of shoots of the overexpressors than those of non-transgenic plants.

Overexpression of *OsWRKY30* in rice plants has been found to enhance drought tolerance (Shen et al., 2012). In contrast, no improved drought tolerance due to overexpression of *OsWRKY30AA*, where all serine residues followed by proline are replaced by alanine residues in the encoded protein, has been observed. The observed interaction of these OsWRKY30 proteins with various MAP kinase proteins suggests that OsWRKY30 functions downstream of the MAPK cascades (Shen et al., 2012). A few studies have reported that overexpression of genes encoding C2H2-type zinc finger transcription factors improves drought tolerance in transgenic rice plants. For example, *ZFP182*-overexpressing transgenic rice plants exhibit increased expression levels of *OsDREB1A*, *OsDREB1B*, *OsP5CS,* and *OsLEA3* and show enhanced tolerance to drought, salinity and low-temperature stresses (Huang et al., 2012), suggesting that ZFP182 may function in the upstream pathway of OsDREB1. Overexpression of *ZFP245* in rice plants has been found to increase tolerance to drought and low-temperature stresses (Huang et al., 2009). Transgenic rice plants display elevated proline levels and ROS-scavenging enzyme activities. Overexpressing *ZFP252* in rice plants leads to enhanced tolerance to drought and salinity stresses and increased proline and soluble sugar contents (Xu et al., 2008). Increased drought tolerance has been observed in transgenic rice plants overexpressing *OsbHLH148*, a gene encoding MeJA-responsive transcription factor (Seo et al., 2011). In the cited study, expression of *OsDREB1* and *OsJAZ* family genes was up-regulated in the overexpressors, and OsJAZ and OsCOI1 proteins were demonstrated to interact with one another. These results suggest that OsbHLH148 acts on the JA signaling cascade with OsJAZ1 and OsCOI1 and functions as an upstream regulator of OsDREB1.

### PROTEIN KINASES

Transgenic rice plants overexpressing *OsCPK4*, a calcium-dependent protein kinase, showed enhanced tolerance to drought and salinity stresses in a study by Campo et al. (2014). In the overexpressors, genes related to lipid metabolism, such as those encoding proteins with lipid binding activities, lipid transfer proteins, and lipases, were up-regulated. Oxidative stress-responsive genes, including peroxidase, thioredoxin, GST, and laccase genes, were also up-regulated in the transgenic plants. These findings suggest that OsCPK4 is involved in the regulation of cellular membrane protection against oxidative damage (Campo et al., 2014). Ho et al. (2013) isolated *OsCDPK1* from sucrose-starved rice suspension cells and developed *OsCDPK1*-overexpressing transgenic rice plants. The transgenic rice plants displayed improved drought tolerance and activated (Ho et al., 2013) expression of a gene for a 14-3-3 protein, GF14c. Transgenic rice plants overexpressing *GF14c* also showed improved drought tolerance, suggesting that enhanced drought tolerance due to OsCDPK1 may be mediated by GF14c. Campo et al. (2012) have reported that transgenic rice plants overexpressing the gene encoding ZmGF14-6, a maize 14-3-3 protein, show enhanced drought tolerance. In their study, expression of stress-responsive genes, including *Rab21* and *Dip1*, was higher under drought stress conditions in transgenic rice plants than in non-transgenic ones, with transgenic plants also exhibiting a higher susceptibility to infection by fungal pathogens. These observations indicate that *ZmGF14-6* functions as a positive regulator in abiotic stress response, but as a negative regulator in biotic stress response. Saijo et al. (2000) discovered that transgenic rice plants overexpressing *OsCDPK7* showed elevated tolerance to drought, salinity, and low-temperature stresses. Overexpression of *OsCDPK7* increased the expression of several stress-responsive genes, suggesting that OsCDPK7 is a positive regulator of abiotic stress response. Finally, transgenic rice plants overexpressing *OsCIPK12* have been found to exhibit enhanced drought tolerance, with increased accumulation of proline and soluble sugars (Xiang et al., 2007).

### RECEPTOR-LIKE KINASES

OsSIK1 is a putative receptor-like kinase (RLK) with extracellular leucine-rich repeats (Ouyang et al., 2010). In the study by Ouyang et al. (2010), transgenic rice plants overexpressing *OsSIK1* showed enhanced tolerance to drought and salinity stresses. Leaves of the transgenic plants exhibited elevated peroxidase, superoxide dismutase and catalase activities and reduced accumulation of H_2_O_2_ compared with those of non-transgenic plants. Reduced stomatal density was also observed in the transgenic plants, suggesting that OsSIK1 may act as a negative regulator for stomatal development. Another rice RLK, OsSIK2, has been reported by Chen et al. (2013). In their study, OsSIK2 was predicted to be an S-domain RLK. Transgenic rice plants overexpressing *OsSIK2* showed enhanced tolerance to drought and salinity stresses, early leaf development, and a delayed dark-induced senescence phenotype. Their results suggest that OsSIK2 is involved in abiotic stress response and senescence processes.

### LEA PROTEINS

Late embryogenesis abundant (LEA) proteins are important stress-inducible proteins involved in cellular protection against stresses (Hanin et al., 2011). Their protective roles in cells include cryoprotective (Bravo et al., 2003) and osmoprotective (Swire-Clark and Marcotte, 1999) behavior to stabilize proteins (Grelet et al., 2005), membranes (Koag et al., 2003), and glassy states (Wolkers et al., 2001). For example, recombinant pea LEA proteins have been shown to protect two mitochondrial matrix enzymes, fumarase, and rhodanese, during drying (Grelet et al., 2005). After transgenically introducing several LEA proteins into rice plants, Hong et al. (1988) investigated stress tolerance in the transgenic rice plants. A barley group-3 LEA protein, HVA1, was specifically accumulated in aleurone layers and embryos at the seed maturation stage. In another study, *HVA1*-overexpressing transgenic rice plants were found to have increased tolerance to drought and salinity stresses, with the increased stress tolerance correlated with HVA1 protein accumulation (Xu et al., 1996). Overexpression of *OsLEA3-1* or *OsLEA3-2* in rice plants leads to enhanced drought tolerance (Xiao et al., 2007; Duan and Cai, 2012). Overexpression of *OsLEA3-2* in yeast improved growth under salinity or osmotic stress conditions, with the OsLEA3-2 protein inhibiting protein aggregation in an *in vitro* assay (Duan and Cai, 2012).

### PHYTOHORMONES

*OsPYL/RCAR5* has been shown to be one of the ABA-signaling components in rice (Kim et al., 2012b). A protein-protein interaction assay and a transient gene expression assay performed by these authors identified an ABA-signaling unit composed of OsPYL/RCAR5, OsPP2C30, SAPK2, and OREB1. Kim et al. (2014) found that overexpression of *OsPYL/RCAR5* induced expression of numerous stress-responsive genes in rice and caused enhanced tolerance to drought and salinity stresses; however, field-grown transgenic rice plants had shorter heights and lower yields than their non-transgenic counterparts. *DSM2* encodes a chloroplast protein, a putative β-carotene hydroxylase involved in biosynthesis of the ABA precursor zeaxanthin (Du et al., 2010). Overexpression of *DSM2* in rice plants enhanced resistance to drought and oxidative stresses and increased xanthophyll levels and non-photochemical quenching.

Zhang et al. (2012) identified and investigated OsPIN3t, a putative auxin eﬄux carrier protein in rice. GFP proteins fused to OsPIN3t were expressed in the plasma membrane, while GUS activity in OsPIN3t promoter-driven GUS transgenic plants was detected in vascular tissues. These subcellular expressions and tissue-specific localization were changed by treatment with auxin transport inhibitors. Transgenic rice plants overexpressing *OsPIN3t* exhibited improved drought tolerance. These results suggest that OsPIN3t regulates polar auxin transport, thereby enhancing drought tolerance.

Isopentenyltransferase (IPT) is an enzyme that mediates cytokinin synthesis. Transgenic tobacco plants expressing the *IPT* gene under the control of a senescence-associated receptor kinase (SAPK), a maturation- and stress-inducible promoter, were developed by Rivero et al. (2007, 2009). The transgenic tobacco plants showed a drastic increase in plant productivity under drought stress conditions. The observed increased plant productivity was attributed to suppression of drought-induced leaf senescence (Rivero et al., 2007) and involvement in photorespiration (Rivero et al., 2009). Similarly, transgenic rice plants expressing the *IPT* gene under the control of the SAPK promoter were generated (Peleg et al., 2011). The developed transgenic rice plants displayed expression changes in genes involved in hormone homeostasis and resource mobilization, a delay in stress responses, and improvement of drought tolerance. In a study by Sun et al. (2014), two rice authentic histidine phosphotransfer proteins (OsAHP1 and OsAHP2) were knocked down simultaneously via RNA interference. The transgenic rice plants showed enhanced tolerance to osmotic stress and hyposensitivity to exogenous cytokinin, suggesting that OsAHPs function as positive regulators of the cytokinin signaling pathway in response to osmotic stress.

### OSMOPROTECTANTS

Ornithine δ-aminotransferase is involved in proline and arginine metabolism. *OsOAT*, a rice gene encoding ornithine δ-aminotransferase, has been identified as a downstream gene of SNAC2 (Hu et al., 2008). You et al. (2012) demonstrated that SNAC2 can bind to the *OsOAT* promoter. In their study, overexpression of the *OsOAT* gene in rice plants enhanced δ-OAT activity and increased proline accumulation, glutachione content, and ROS-scavenging enzyme activity. The *OsOAT*-overexpressing transgenic rice plants displayed enhanced oxidative, drought, and osmotic stress tolerance. While seedling shoot lengths were similar between transgenic and non-transgenic plants under normal conditions, reduced inhibition of shoot growth was observed in transgenic plants under osmotic stress conditions compared with non-transgenic plants.

*OsTPS1*, a gene encoding a rice trehalose-6-phosphate synthase, acts as a key enzyme for trehalose biosynthesis. Overexpression of the gene in rice plants improved tolerance to drought, salinity, and low-temperature stresses in an investigation by Li et al. (2011). In the transgenic rice plants, trehalose and proline contents were increased and some stress-responsive genes, including *WSI18*, were up-regulated relative to those in non-transgenic plants.

### OTHER GENES

Other genes encoding proteins with various characteristics have also been shown to enhance drought tolerance. Some of these proteins are stress responsive. The effect of overexpression of *O. sativa Drought-Induced LTP* (*OsDIL*), a lipid transfer protein gene, on drought stress tolerance in rice was investigated by Guo et al. (2013). The transgenic plants showed increased tolerance to drought stress at both vegetative and reproductive stages. Less severe tapetal defects and fewer defective anther sacs were observed in the transgenic plants. These results were consistent with data indicating that the *OsDIL* gene is expressed in anthers. Overexpression of the heat shock protein gene *OsHsp17.0*, or *OsHsp23.7*, has been found to improve tolerance to drought and salinity stresses in rice (Zou et al., 2012). In that study, the transgenic rice plants had lower relative electrical conductivities and malondialdehyde contents and higher proline contents compared with non-transgenic rice plants.

Modulation of ROS accumulation is also important for the enhancement of drought tolerance. Transgenic rice plants overexpressing the gene encoding manganese superoxide dismutase, an antioxidant enzyme, have improved osmotic stress tolerance (Wang et al., 2005). The cited authors found that electrolyte leakage in the transgenic plants was lower than in non-transgenic plants under osmotic stress conditions, and that photosynthetic rate was less affected by osmotic stress in the transgenic plants. The enzyme OsMIOX, a rice *myo*-inositol oxygenase, catalyzes the oxidation of myo-inositol to glucuronic acid. In a study by Duan et al. (2012), the *OsMIOX* gene was overexpressed in rice plants and caused increases in ROS-scavenging enzyme activities and proline content and enhancement of growth performance under osmotic stress conditions. Ski-interacting protein (SKIP), identified by yeast two-hybrid screening using the avian retrovirus oncogene v-Ski as bait (Dahl et al., 1998), has been well characterized as a transcriptional coregulator and a spliceosome component in humans (Figueroa and Hayman, 2004). Transgenic rice plants overexpressing *OsSKIPa* have shown improved drought tolerance and increased ROS-scavenging ability. Higher transcript levels of *SNAC1*, *OsCBF2*, *OsPP2C*, and *OsRD22* have been found in *OsSKIPa*-transformed rice plants compared with their non-transgenic counterparts (Hou et al., 2009).

Protein turnover via ubiquitin-dependent protein degradation and ribosomal protein synthesis has been shown to be involved in abiotic stress response regulatory networks. OsSDIR1 (*O. sativa* SALT-AND DROUGHT-INDUCED RING FINGER 1) is a functional RING-finger-containing E3 ligase, with the RING finger region required for its activity (Gao et al., 2011). Transgenic rice plants overexpressing *OsSDIR1* show enhanced drought tolerance and stomatal closure (Gao et al., 2011), while those overexpressing *OsRDCP1*, a rice RING domain-containing protein 1 gene, have improved drought tolerance (Bae et al., 2011). Molecular mechanisms underlying the improved drought tolerance of these transgenic rice plants remain largely unclear. Jiang et al. (2012) observed that transgenic rice plants overexpressing *OSRIP18*, a rice ribosome-inactivating protein 18 gene, exhibited improved drought and salinity tolerance. Microarray analysis detected differentially expressed genes, most of which were not regulated by abiotic stresses, in the transgenic rice plants.

RNA turnover may also be involved in abiotic stress-response regulatory networks. *OsSUV3* encodes an NTP-dependent RNA/DNA helicase (Tuteja et al., 2013). Transgenic rice plants overexpressing *OsSUV3* show reduced lipid peroxidation, electrolyte leakage, and H_2_O_2_ production, and enhanced antioxidant enzyme activities, thereby leading to enhanced tolerance to osmotic and salinity stresses (Tuteja et al., 2013). GRP encodes a glycine-rich RNA-binding protein. In a study by Yang et al. (2014), transgenic rice plants overexpressing *Arabidopsis GRP2* or *GRP7* displayed increased grain yield under drought conditions. The increased grain yield was caused by improved grain filling. Several stress-responsive genes, including *OSE2*, *Dip1*, and *PBZ1*, were up-regulated in the transgenic rice plants.

The application of genes encoding metabolic enzymes is also thought to be useful for the enhancement of drought tolerance. Squalene synthase (SQS) is one of several farnesyl-diphosphate farnesyltransferase proteins that catalyze the first reaction of the branch of the isoprenoid metabolic pathway involved in sterol biosynthesis (Tansley and Shechter, 2001). Disruption of *SQS* gene function by RNA interference has been found to improve drought tolerance in rice plants, with the transgenic plants showing increased root length, an elevated number of lateral roots and reduced stomatal conductance (Manavalan et al., 2012). Polyamines, such as putrescine, spermidine, and spermine, are compounds implicated in plant embryo development, stem elongation and stress response (Takahashi and Kakehi, 2010). Polyamine levels can be modulated by the regulation of metabolic enzymes, including arginine decarboxylase. Because rice plants overexpressing *Datura stramonium* arginine decarboxylase show improved drought tolerance along with increased putrescine content, Capell et al. (2004) have proposed a regulatory mechanism linking putrescine metabolism to drought tolerance. In contrast to putrescine, spermidine, and spermine are not involved in drought stress tolerance, as increased spermidine and spermine content has not been observed to enhance drought tolerance in rice plants (Peremarti et al., 2009).

Transgenes originating from non-plant species have also been used to enhance stress tolerance. Harpin proteins are secreted by the type-III protein secretion system of Gram-negative plant pathogenic bacteria (Wei et al., 1992). Harpin proteins trigger the hypersensitive response, a well-characterized defense response against various bacteria, fungi, nematodes, and viruses. Transgenic rice plants overexpressing the harpin-encoding gene hrf1 showed improved drought tolerance along with increased stomatal clousure and ABA, proline, and soluble sugar contents (Zhang et al., 2011). Increased expression of stress-responsive genes including *OsLEA3-1* was also observed in the transgenic rice plants. As reviewed by Sharma et al. (2013), the evidence that pathogenic-related genes can also improve abiotic stress tolerance suggests an overlapping regulatory cascade between biotic and abiotic stresses.

## CHANGES IN SHOOT GROWTH OF DROUGHT-TOLERANT TRANSGENIC RICE PLANTS

Among the transgenic rice plants described in this review, 37% have been reported to display growth retardation under normal conditions or exogenous ABA application (**Table 1**). Such decreased shoot growth performance is also observed in non-transgenic plants subjected to drought stress conditions. Shoot growth retardation due to low soil water content is one of the earliest stress responses in plants, occurring even earlier than decreases in leaf water potential (Michelena and Boyer, 1982; Parent et al., 2010). This phenomenon suggests that plants actively decrease shoot growth instead of it being a consequence of decreased cell turgor (Claeys and Inzé, 2013). Growth regulation in proportion to soil water content is thus an important plant morphological response to water deficit. Molecular mechanisms underlying growth regulatory responses to water deficit have been investigated in *Arabidopsis*. DELLA proteins, which are negative regulators of gibberellic acid (GA) signaling, have been shown to integrate growth and abiotic stress tolerance in *Arabidopsis* (Achard et al., 2006). Skirycz et al. (2010) performed transcript profiling of expanding *Arabidopsis* leaves subjected to mild osmotic stress. Their results indicated that an ethylene- and gibberellin-dependent regulatory circuit modulated growth under the mild osmotic stress conditions, with no involvement from ABA. Rapid accumulation of 1-aminocyclopropane-1-carboxylic acid (1-ACC), an ethylene precursor, was observed in the expanding leaf tissue under the mild osmotic stress conditions of their study. This accumulation has been proposed to activate a cascade of the growth regulatory circuit in *Arabidopsis* as follows (Claeys and Inzé, 2013). After activation by 1-ACC accumulation through a MAP kinase cascade, ethylene responsive factor 6 (ERF6) increases expression of *GA2OX6*, which encodes an enzyme that inactivates GAs. By the operation of GA2OX6, GAs are inactivated, with this inactivation stabilizing DELLA proteins. The DELLA proteins modulate the activity of ANAPHASE-PROMOTING COMPLEX/CYCLOSOME (APC/C), which controls the activity of CDK-cyclin complexes, through the repression of APC/C inhibitors DEL1 and UVI4. Finally, the modulated APC/C activity abolishes potential for cell proliferation and inhibits growth.

A similar growth regulatory circuit does not seem to hold for rice, as it has been generally accepted that ethylene and 1-ACC act as positive growth regulators under various conditions in rice (Bailey-Serres and Voesenek, 2008, 2010; Fukao and Xiong, 2013; Wang et al., 2013). In rice, growth regulatory mechanisms that are distinct from those in *Arabidopsis* should therefore be taken into consideration. We recently identified *O. sativa phytochrome interacting factor like 1* (*OsPIL1)*, a gene that regulates internode elongation under drought stress conditions in rice (Todaka et al., 2012). The bHLH-type transcription factor OsPIL1 functions as a transcriptional activator and modulates expression of cell elongation-related genes such as expansins. Increased expression of *OsPIL1* observed in the daytime under normal growth conditions was canceled under drought stress conditions. We proposed the following growth regulatory mechanistic model involving OsPIL1 in response to drought stress. Under normal growth conditions, OsPIL1 elevates expression of cell elongation-related genes such as expansins, causing normal shoot growth. When rice plants are exposed to drought stress, the canceled OsPIL1 expression leads to reduced expression of cell elongation-related genes, resulting in shoot growth reduction that likely conserves photosynthetic products and decreased shoot surface area. The saved energy may be used for activation of mechanisms involved in stress tolerance. Ogawa et al. (2011) have revealed that the rice protein RSS1 plays an important role in the maintenance of meristematic activity under salinity stress conditions. RSS1 proteins interact with protein phosphatase 1, a regulator of various cellular processes including the cell division cycle. RSS1 stability is regulated by the APC/C 26S proteasome pathway, which is responsible for degradation of mitotic cyclins.

Use of the growth regulatory genes described in this section may ameliorate the growth reduction observed in drought-tolerant transgenic rice plants. Although stress-inducible promoters are often useful for development, their efficacy seems to be limited under moderate long-term drought stress conditions. Long-term drought stress maintains a high level of transgene expression, thereby affecting growth performance.

## EVALUATION OF ABIOTIC STRESS TOLERANCE UNDER FIELD CONDITIONS

When developing abiotic stress tolerant transgenic crops, plant productivity should be taken into consideration. Plant productivity is extensively affected by natural drought episodes under field conditions. Droughts are unpredictable events and vary in stress severity and duration. Simultaneously occurring stresses, such as drought and heat, are also observed. Results obtained under laboratory or greenhouse conditions are therefore not perfectly comparable to observations made under field conditions. Field trials are thus critical for the proper evaluation of stress-tolerant transgenic crops.

Xiao et al. (2007) analyzed drought tolerance of transgenic rice plants constitutively overexpressing *OsLEA3-1*, a gene encoding proteins that highly accumulate in water-stressed tissues, as well as plants expressing the transgene with a stress-inducible promoter under field conditions. Drought stress was initiated at the panicle development stage by draining surface water in paddy fields and halting irrigation until leaves were rolled. Although T_1_ generations of both transgenic lines showed reduced yields under non-stressed conditions, T_2_ and T_3_ generations exhibited no yield penalty under non-stressed conditions and exhibited increased grain yield under drought conditions.

Xiao et al. (2009) also examined drought tolerance of transgenic rice plants overexpressing seven well-documented stress-related genes with an actin promoter under field conditions. These seven genes were *CBF3/DREB1A*, an AP2/ERF-type transcription factor; *SOS2*, a serine/threonine protein kinase; *NCED2* and *LOS5*, enzymes involved in ABA biosynthesis; *NPK1*, a mitogen-activated protein kinase kinase kinase; *ZAT10*, a C2H2-type zinc finger transcription factor; and *NHX1*, a vacuolar Na^+^/H^+^ antiporter. Although drought stress in the field decreased grain yield in these transgenic plants, grain yields in *LOS5*, *ZAT10*, and *NHX1* overexpressors were less affected. The authors also developed transgenic rice plants that expressed these genes with a stress-inducible promoter and field-tested their drought tolerance. Grain yields in these transgenic plants were similarly decreased by drought stress under field conditions. Grain yields in transgenic rice plants expressing *CBF3/DREB1A*, *SOS2*, *NPK1*, *LOS5*, *ZAT10*, and *NHX1* with the stress-inducible promoter were the least affected. Because absolute grain yields under normal growth conditions were lower in these transgenic rice plants than in non-transgenic ones, further improvement is needed for practical application.

Hu et al. (2006) subjected field-grown transgenic rice plants overexpressing *SNAC1*, a NAC-type transcription factor, to two different levels of drought stress treatments at the anthesis stage: severe stress with 15% soil moisture and moderate stress with 28% soil moisture. Both drought stress conditions increased spikelet fertility in the transgenic plants. Under non-stressed conditions, agronomic traits, including plant height, panicle number, spikelet number, spikelet fertility, and grain yield, were similar between transgenic plants and the controls. Drought resistance of transgenic rice plants overexpressing OsNAC5 (Jeong et al., 2013), OsNAC9/SNAC1 (Redillas et al., 2012), or OsNAC10 (Jeong et al., 2010) under the control of the root-specific promoter has also been examined in the field. In these studies, exposure to drought stress was performed at the panicle heading stage by draining surface water and halting irrigation until leaves were rolled. Similar results were observed among the three transgenic rice lines. Grain yield decreases under drought conditions were significantly smaller in all three transgenic lines than those observed in their non-transgenic counterparts. Drought tolerance of transgenic rice plants overexpressing *OsOAT* has also been investigated under field conditions (You et al., 2012). The OsOAT protein, an enzyme that increases proline content, is a direct target gene of the stress-responsive NAC transcription factor SNAC2. The field drought test was performed by stopping irrigation at the flowering stage in a refined paddy field covered with a movable rain-off shelter. Slower wilting, fewer withered leaves, and a higher rate of seed-setting were noted in the transgenic rice plants than in non-transgenic ones.

Increased grain yield was observed in transgenic rice plants overexpressing the *AP37* gene, an AP2/ERF-type transcription factor, when the transgenic plants were subjected to drought stress in the field (Oh et al., 2009). The field drought stress was performed at the panicle heading stage by draining the surface water and halting irrigation until leaves were rolled. The increased grain yield was due to the higher grain-filling rate in the drought-treated transgenic plants compared with the drought-treated non-transgenic plants. Finally, field evaluation of transgenic rice plants overexpressing *EDT1/HDG11*, a homeodomain-leucine zipper transcription factor, has also been carried out (Yu et al., 2013). The transgenic rice plants were grown in the field for a month after transplanting; irrigation was then stopped until the seed maturation stage. The drought-treated transgenic rice plants had higher grain yields than those observed in the drought-treated non-transgenic rice plants. The grain yield increase in the transgenic plants was a consequence of their larger panicle sizes and higher tiller numbers compared with the non-transgenic plants.

## FUTURE DIRECTIONS IN THE DEVELOPMENT OF DROUGHT-TOLERANT TRANSGENIC RICE PLANTS

Although several studies have reported transgenic rice plants with improved drought tolerance during field trials, further research is needed to uncover the regulatory mechanism of drought response and tolerance under field conditions. Such investigations should lead to the discovery of new genes that increase drought tolerance without yield penalty even under drought conditions. Another approach to new gene exploration is to study stress tolerance mechanisms of stress-adapted extremophiles such as desert plants, halophilic plants, cold-water fishes, and thermophilic bacteria (Mittler and Blumwald, 2010). Even in well-characterized species, the functions of 18–38% of total proteins remain unknown (Gollery et al., 2006). The elucidation of these unknown function proteins should aid the discovery of new genes. Modification of root architecture is also important for the development of drought-tolerant rice plants. In this regard, Uga et al. (2013) reported that the QTL *Deeper Rooting 1 (DRO1)* increased the root growth angle in rice, leading to high-yield performance under drought conditions.

Rice has the highest potential of any crop to grow under submergence conditions. Studies of submergence-tolerance mechanisms and the development of submergence-tolerant rice cultivars have progressed significantly (Bailey-Serres and Voesenek, 2008, 2010; Hattori et al., 2009; Fukao and Xiong, 2013; Voesenek and Bailey-Serres, 2013). The results of these studies indicate that drought-tolerant rice plants with submergence-tolerant cultivar backgrounds are exceptional crops that can survive under both low and excessive soil–water content conditions. In the future, crops may be alternately exposed to drought and flood because of global climate change. Efforts to develop rice cultivars having high water usage flexibility should help solve this crisis.

## AUTHOR CONTRIBUTIONS

DT wrote all sections of the manuscript with the assistance of KS and KYS.

## Conflict of Interest Statement

The authors declare that the research was conducted in the absence of any commercial or financial relationships that could be construed as a potential conflict of interest.
